# Micropoetry Meets Neurocognitive Poetics: Influence of Associations on the Reception of Poetry

**DOI:** 10.3389/fpsyg.2021.737756

**Published:** 2021-10-20

**Authors:** Katharina Gloria Hugentobler, Jana Lüdtke

**Affiliations:** Department of Education and Psychology, Experimental and Neurocognitive Psychology, Freie Universität Berlin, Berlin, Germany

**Keywords:** neurocognitive poetics, associations, literary reading, text comprehension, computational linguistics

## Abstract

Reading and understanding poetic texts is often described as an interactive process influenced by the words and phrases building the poems and all associations and images induced by them in the readers mind. Iser, for example, described the understanding process as the closing of a good Gestalt promoted by mental images. Here, we investigate the effect that semantic cohesion, that is the internal connection of a list words, has on understanding and appreciation of poetic texts. To do this, word lists are presented as modern micropoems to the participants and the (ease of) extraction of underlying concepts as well as the affective and aesthetic responses are implicitly and explicitly measured. We found that a unifying concept is found more easily and unifying concepts vary significantly less between participants when the words composing a micropoem are semantically related. Moreover these items are liked better and are understood more easily. Our study shows evidence for the assumed relationship between building spontaneous associations, forming mental imagery, and understanding and appreciation of poetic texts. In addition, we introduced a new method well-suited to manipulate backgrounding features independently of foregrounding features which allows to disentangle the effects of both on poetry reception.

## 1. Introduction

*The text, we have seen, patterns and delimits, but it ultimately functions like a chemical element: it itself is merged in the synthesis with the other elements to produce a particular event-a poem*.

– Louise M. Rosenblatt

We, as a universal society, have long agreed that a poem is more than a mere collection of words: it is, we might feel, a picture painted with words. Psycholinguistic and neurocognitive research has only just started to explore the underlying cognitive and affective processes which help us make sense of a poetic text (Jacobs and Kinder, 2015; Jacobs and Willems, 2018) and ultimately shape and form mental images often reported while reading poetry (Belfi et al., 2018; Papp-Zipernovszky et al., 2021).

As already assumed by proponents of the readers-response theories like Iser (1978) and Rosenblatt (1978), reading and especially reading literature can only be understood as an interaction between features of the text and characteristics of the reader, and, as recently highlighted, also by the task at hand and the reading situation (Jacobs, 2015a; Bohn-Gettler, 2019). As described in the Transactional Theory by Rosenblatt (1978), reading of a poetic text is a process of meaning construction in which the linguistic symbols, the words and the written or printed marks have to be distinguished from what a listener or reader evokes from them (Rosenblatt, 1978). Rosenblatt used the term poem only for the later one. Iser described the meaning construction process as “one of the activities through which we form the ‘gestalt' of a literary text” based on “the ‘picturing' that is done by our imagination” (Iser, 1972, p. 288). But how does this interaction between reader and text come about? Let us start our analysis by looking at the interaction between text and reader from afar: At low resolution the emphasis is on the influence of literature genre on reading behaviour. According to proponents of the Reader Response Theory, reading behaviour depends on the reader's assumptions about the text (Hanauer, 1998; Topping, 2015) and a reading mode relevant to the understanding of the genre is adopted according to the genre choice made. As choices and adaptation of reading mode take time, several empirical studies demonstrated that texts labelled as factual are read faster compared to fictional texts (Zwaan, 1994; Wolfe, 2005; Altmann et al., 2014, see Hartung et al., 2017, for contrasting results). Also memory for individual aspects of a text differs as a function of the type of text (Zwaan, 1994). In line with the general assumption that classification of a text as a poem gives rise to a reading mode characterised by heightened awareness of language patterns and hidden meanings (for a comprehensive overview see Hanauer, 1998 and Carminati et al., 2006). Osowiecka and Kolanczyk (2018) found that poetry reading stimulates divergent thinking and vice versa.

According to Graesser (2015), the above theories are sufficient to explain basic levels of comprehension, but they are insufficient to describe deeper comprehension processes assumed to play a role when reading literature. The latter requires to also consider parameters such as the influence of emotions, situations etc. both through the literal content in itself, but also through an interaction between reader and text. In other words, text-bound variables like rhythm (Kentner, 2012; Peelle and Davis, 2012), prosody (Paulmann et al., 2013), valence and arousal within the words employed (Kuchinke et al., 2005; Warriner et al., 2013; Jacobs et al., 2016; Sylvester et al., 2016) and reader-centered variables such as affective and aesthetic responses (De Jaegher and Di Paolo, 2007; Gallagher, 2012; Winkielman et al., 2014), often studied from an embodied perspective, are closely intertwined. The Neurocognitive Poetics Model (NCPM; Jacobs, 2011, 2015a,c; Jacobs and Kinder, 2017) aims at integrating data on neurocognitive and emotional (Jakobson, 1960; Panksepp, 2008) processes during literature comprehension. It is conceptualised as a dual-process model which is informed by Gestalt theory and as such distinguishes between back- and foreground elements. The understanding of a text is conceived as successful closing of a good Gestalt. Within the framework of the model a text consists of familiarising background elements which cater to the readers need for coherence and meaning construction. The model further assumes that backgrounding features are stronger associated with immersive reactions (Van Krieken et al., 2015) and a mood-empathic reading mode (Lüdtke et al., 2014; Jacobs et al., 2016). Emotional and aesthetic feelings (Fitch, 2009; Westphal-Fitch and Fitch, 2018) are created against this background through foregrounding text elements. Foregrounding elements have a defamiliarising effect (Kuiken et al., 2012; Jacobs, 2015a,b,c) thereby eliciting what Mukarovský (1964) described as a de-automatisation in the reading process, leading to reduced reading speed as a result of more and intensive active drawing of inferences. Although manipulation of words always influences both backgrounding and foregrounding elements, empirical studies on reading poetic texts primarily focussed on the manipulation of foregrounding elements. Hakemulder (2004) for example changed isolated lines of poems to manipulate the amount of foreground features and observed that foregrounding caused higher aesthetic appreciation. Obermeier et al. (2013) manipulated rhyme and metre and also demonstrated that both foregrounding features enhanced aesthetic appreciation. As a text is characterised by both background and foreground features, experimental studies on the influence of background features like word frequency are equally important for the study of poetry comprehension. Yet studies focussing on the isolated manipulation of background features are few and far between (Hanauer, 1998). This raises the question of whether background features have any influence on comprehension of poetic texts at all? The short answer is: they do. For the long answer we need to look at studies addressing these elements.

Facilitation or ease of processing, i.e., the ease of mental operations concerned with stimulus meaning and its relation to semantic knowledge structures, is known to affect perceived aesthetic value. This is recognised as the mere-exposure effect, which links repetition of a stimulus to enhanced liking for this initially neutral stimulus (Zajonc, 2000). In support of this idea, Menninghaus et al. (2017) showed that parallelistic diction, a supra-lexical background feature, enhances emotional and aesthetic appreciation as well as semantic processing. In a similar way Reber and Schwarz linked variables that facilitate the processing of a stimulus (e.g., priming, mastery and the amount of information) to positive affective reactions (Reber et al., 2002, 2004). Markedly, aesthetic appreciation, a bona fide reaction to the presence of foregrounding elements, is reciprocally influenced by and at the same time acts as a positive influence on processing fluency (Reber et al., 2004). Moreover, the absence of prototypical narrative features (which are often discussed as an example of backgrounding features Hakemulder, 2020) strongly increases processing efforts as measured by reading times (Castiglione, 2017). Manipulation of word frequency, perhaps the most prototypical backgrounding feature, is widely employed to highlight the influence of facilitating factors on processing speed and accuracy (Murray and Forster, 2004) and appears to mediate pleasentness- and familiarity ratings on single word level (Sluckin et al., 1980).

Although ease of processing was shown to influence the above, the concept is not sufficient to explain the closing of a good Gestalt *sensu* (Iser, 1972). While an explanation based on the ease of processing focusses on single word level cognition (e.g., higher word frequency leads to faster word recognition), a good Gestalt results from the integration of words. In the same way that cognitive theories assume that associations are the most important principle for organisation of our semantic knowledge (for an overview cf. Hofmann and Jacobs, 2014), they should also guide the closing of a good Gestalt while reading poetic texts. In this regard, we can posit an influence of what James (cited after Mangan, 2001, 2008) described as the fringe of consciousness on processing fluency-mediated construction of meaning: It represents context information in consciousness, conveys a sense of rightness and brings out the notion that more information is available.

What do we already know about the role of associations on text comprehension processes related to the closing of a good Gestalt? According to the Gestalt principle of closure, objects which are grouped together are perceived as a whole which might otherwise (i.e., if the objects were regarded as individual entities) not exist and which is more than or different from the combination of its parts. The emergence of a resulting Gestalt from an albeit sketchy figure is hinged on the presence of a unifying pattern (Warriner, 1923). Wolfgang Iser transmitted that principle onto the process of reading literary texts and therefore poetry: “By grouping together the written parts of the text, we enable them to interact, we observe the direction in which they are leading us, and we project onto them the consistency which we, as readers, require. This ‘gestalt' must inevitably be colored by our own characteristic selection process” (Iser, 1972, p. 289). A poem, composed of words that interrelate, facilitates the application of the grouping principle of closure so that a good Gestalt can emerge and potentially foster the creation of a mental image (Iser, 1972).

Yet, words have an effect on comprehension in their own right. Readers pre-activate concepts beyond the unfolding sentence to simulate the described event for predictive (online) language comprehension (for an overview see Huettig, 2015). Associations or mental connections between concepts or events are by some described as the product of contiguity, i.e., the probability of co-occurrence between words. Others conceptualise associations as the result of an event simulation heuristic which pre-activates linguistic representations (Huettig, 2015). Semantic cohesion serves as a measure for the strength of internal association between concepts and was shown to influence sentence processing on single sentence level (Hintz et al., 2020). Current machine-learning methodologies (Grainger and Jacobs, 1996; Dijkstra and van Heuven, 2002; Hofmann and Jacobs, 2014; Jacobs, 2018) as well as some association databases rely on contiguity-based approaches. Accordingly, in a recent study Jacobs and Kinder showed that latent semantic analysis (LSA, a method based on distributional semantics to predict upcoming words) is superior to surface model analysis for the prediction of ratings on difficulty and aesthetic liking of metaphors (Jacobs and Kinder, 2017). This reveals the power of associative databases which exceed purely systematic relations (such as WordNet) and instead rely on a rich network of semantic relations informed by cultural, emotional and personal experience (Netzer et al., 2009).

Poems lend themselves to the study of neurocognitive processes during reading, seen as they are well constrained literary material (Müller et al., 2017). Seen in the light of associative networks, poetry appears to be characterised by lexical associations (Netzer et al., 2009). Correspondingly, a computational analysis of poems written by professional and amateur poets revealed that the former use more concrete words to create a canvas onto which readers can project their associations. Professional poets avoid direct references to abstract and intangible concepts and instead let images instead of words convey emotions, concepts, and experiences (Kao and Jurafsky, 2012).

**Figure 1:**
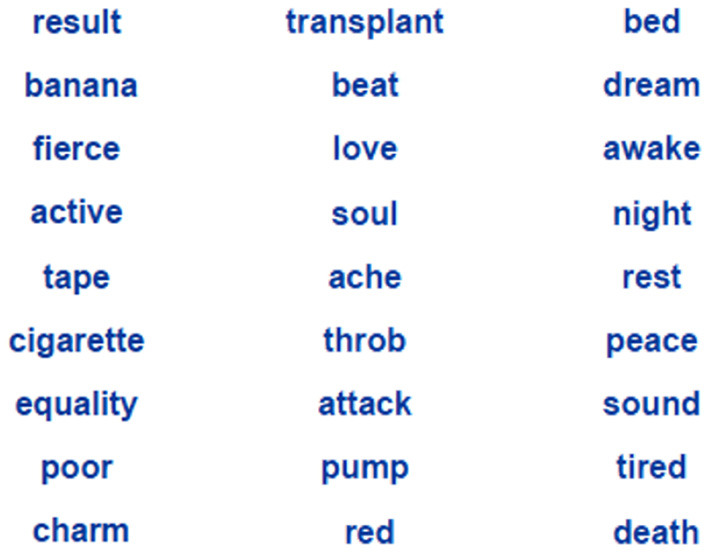
Three examples of items presented as micropoems in this study. Items are ordered according to their semantic cohesion (in parenthesis), from left to right: Match 4 (0.208), EAT 3 (0.264), EAT 5 (0.369). For a comprehensive overview over the items please cf. **Table 1**; Supplementary Tables 1, 2.

With the above in mind, we want to investigate the effect of associations, as an example of a backgrounding feature, on the closing of a good Gestalt and maintain that the effect exceeds a mere effect of facilitated processing. To test this proposition, we created simple word lists, which are essentially devoid of foreground features, and presented them as versions of poetic texts (Figure 1). The participants are asked to read lists of nine words with varying degree of semantic cohesion introduced as micropoems (Jacobs, 2015b). They are then asked to produce a possible one-word-title for each poem and rate the micropoems on dimensions Comprehensibility, Liking, Imageability as indication of reader's response at both the upper and lower route according to the NCPM (Jacobs, 2015a). This setup allows for an analysis of the influence of background features -more precisely the role of semantic cohesion and word frequency in the present case- on the closing of a good Gestalt independent of the influence of foregrounding figures.

## 2. Hypotheses

As each and every word is linked to a plethora of other concepts, reading such a simple word list not only evokes simulation of the mentioned concepts but also co-activates the concepts associated with those words (Mangan, 2001; Hofmann and Jacobs, 2014). Each group of concepts associated with a single word in itself is likely to evoke its own association, seen as they are all semantically related and trace back to a common anchor word (Deese, 1959; Roediger and McDermott, 1995). The idea behind our set-up is *not* that each word *per se* leads to the generation of many associations. Instead, the words taken together are all highly semantically related (high semantic cohesion as a feature facilitating formation of a Gestalt) and thus, as a whole, facilitate the generation of associations in the reader's mind. We therefore assume a reciprocal enhancement of activation which facilitates the formation of a consistent mental image or good Gestalt as for example opposed to a serial position effect. If only the first or the last word of a poem was used for the generation of a title, we should not observe any effect of semantic cohesion on semantic relatedness. Reading poems with low internal semantic cohesion, on the other hand should merely activate concepts vaguely associated with the individual words. What is more, associations may neutralise each other through lateral inhibition at the conceptual level (for a thorough analysis see Hofmann and Jacobs, 2014) and therefore inevitably fail to close a good Gestalt.

Consequently, we should be able to test whether semantic cohesion of the words composing a poem has a significant impact on the closing of a good Gestalt, and therefore of the formation of mental imagery, a process associated with the recognition of the bigger picture conveyed by a poetic text. Hence, we expect participants to understand the micropoems with higher semantic cohesion more easily and additionally report the experience of more mental imagery. Moreover, semantic cohesion should also influence affective and aesthetic responses as described in the NCPM (Jacobs et al., 2015). Following the scheme introduced by Dixon and Bortolussi (2015) a set of explicitly surveyed rating data (mood induced, liking of the micropoem, perceived difficulty of the micropoem, perceived imagery induced) and ancillary reaction parameters (mean semantic relatedness of the response, mean reading time) as implicit control variables will be used.

In accordance with the assumptions of the NCPM, a modification of micropoems in terms of internal semantic cohesion should be reflected in the mood induced in the reader (NCPM: backgrounding, fast route) and self-reported aesthetic appreciation of the micropoems (NCPM: foregrounding, slow route) (Jacobs, 2015a). With regards to difficulty, the effect should be mirrored in the self-reported difficulty to understand the poems (explicit measure) and reading times (implicit measure). The capacity of a poem to induce a mental image in the reader should be seen in a self-reported rating of perceived mental images. Moreover, participants will be asked to put their first spontaneous association to the poems forward in form of a freely chosen title. Semantic relatedness between spontaneous associations to the poems (i.e., titles) and the words composing a poem as well as the number of different titles found, as an indicator for the closing of a good Gestalt, are used to control this effect with an implicit measure.

One might argue that the effects discussed above can be put down to facilitated processing due to semantic cohesion. Following this line of thought, if the root cause of any differences observed was indeed attributable to an ease of processing, this effect should reproduce if another factor influencing the ease of processing (e.g., supra-lexical word frequency) was modified. If supra-lexical word frequency has no or a different effect, then it is clear that the influence of semantic cohesion is based on more than a simple facilitation of word identification.

We expect self-reported mood and imagery to be stronger for micropoems with high semantic cohesion than low semantic cohesion. Micropoems with high semantic cohesion should be easier to understand (less self reported difficulty in understanding) and should be better liked. Regarding the implicit measures we expect higher semantic relatedness between the title and the reported associations (i.e., titles given) and faster reading times for micropoems with high internal semantic cohesion. Moreover the intra-subject variance of associations found should increase for micropoems with reduced internal semantic cohesion.

We attribute the effects to the evolution of more elaborate associations in the participants' minds as a function of internal semantic cohesion and therefore do not expect any effect for word frequency.

## 3. Methods

### 3.1. Participants

The sample was composed of 32 participants (20 female; age: *M* = 46.13, *SD* = 13.96, range = 24–74) all of whom learned English as their first language. Participants were recruited through an online survey employing the www.soscisurvey.de platform. A total of 9 participants reported to have been exposed to a language other than English before the age of 5, while 27 of 32 participants lived in an English speaking environment at the time of the survey and 28 participants used English as their most frequent language. The experiment followed the rules set by the ethical guidelines of the German Psychological Society's 121 (DGPs, 2004, CIII). Participants were informed about taking part in research, about the possibility of quitting the experiment with no disadvantage at any time and about the fact that all data were collected and analysed anonymously. They provided informed consent and gave permission to use their collected data anonymously for publications.

#### 3.1.1. Inclusion Criteria

The sample was stratified by first language (BE/AE/undefined), second language (yes/no), current immersion in English speaking environment (yes/no) and English as dominant language (yes/no). Participants who spoke any language other than English as their first language were excluded by default (*n* = 6). Data quality was checked based on the mean semantic distances obtained or the titles produced by the participants and the words constituting a poem (for calculation, please see section 3.4.2). If a participant had entered titles at random the corresponding mean semantic distance of their answers should differ from the global mean. We did not observe such behaviour in the data, therefore all remaining *n* = 32 (after exclusion of *n* = 6 non-native speakers) participants were included into the final dataset. This constitutes a 84.21% of the original *n* = 38 sample.

### 3.2. Item Design

We created a first set of micropoems with high semantic similarity, which consisted of nine words each. The micropoems were drawn as subsamples of the Edinburgh Associative Thesaurus, a database for 8,400 stimulus words together with their most frequent word associations, which were collected in an empirical free-association set-up based on 100 subjects (Kiss et al., 1973).

To select a set of six micropoems the following criteria were used. We first selected only those stimulus words from the original EAT for which nine different word associations (maximum number of associations reported) were listed. We further reduced the list of stimulus words by excluding all stimulus words with missing values for word frequency (taken from the SUBTLEX-UK, van Heuven et al., 2014) and word-based valence, arousal, concreteness and imagery (taken from Bestgen and Vincze, 2012) for the nine word associations. For the resulting list of stimulus words we calculated the supra-lexical values for the associated micropoems by calculating the mean values for word-based valence, arousal, concreteness and imagery for the nine word associations constituting each micropoem. We then excluded all stimulus words and associated micropoems with supra-lexical valence, arousal and concreteness values outside the global interquartile range (IQR) of the dimensions valence [4.2, 6.1] and arousal [4.82, 5.36] calculated for all words reported by Bestgen and Vincze (2012).

To manipulate the second predictor, the supra-lexical level of word frequency, we looked for micropoems with high and low supra-lexical word frequency. Although the correlation between word frequency and word imageability is in general very low (Stadthagen-Gonzalez and Davis, 2006), we explicitly tried to avoid any confoundation between word frequency and imageability. Consequently, we first used the supra-lexical imagery for the associated micropoems of the remaining stimulus words and divided them in three groups: a low imagery, a medium imagery and a high imagery one. For doing so we used the first quartile, the IQR, and third quartile for word imagery of the Bestgen and Vincze list as defining criteria. In a final step we chose poems with a supra-lexical word frequency above, within, and below the IQR of lexical word frequency taken from SUBTLEX-UK [2092; 33275].

We controlled the respective numerical semantic cohesion (cf. section 3.3 below) and found that although the micropoems were composed of highly associated words (determined in a free association set-up), they show substantial differences between empirical semantic association and numerical semantic cohesion. Therefore, in a second step we constructed six new micropoems constituted of a list of nine words with no or even low semantic associations and controlled for semantic cohesion as well as independence of semantic cohesion and word frequency. To make sure, that the six EAT-based micropoems (EAT-1 to EAT-6) and the six matching micropoems (Match-1 to Match-6) do not differ with respect to the supra-lexical values of interest, we used the program Match (Van Casteren and Davis, 2006) to randomly pick nine words matching the nine words constituting the EAT-based micropoems on dimensions valence, arousal, imagery, concreteness, frequency, and word length. The drawing pool for the words constituting the Match-based micropoems consisted of all stimulus words of the EAT for which all psycholinguistic values mentioned above were available. Words already part of one of the six EAT-based micropoems and words, that were both noun and verb (e.g., “fight”), were excluded.^1^ The resulting twelve EAT-based and Match-based micropoems as well as their relevant measures at the lexical level are reported in Supplementary Tables 1, 2. The supra-lexical values for all micropoems are summarised in Table 1.

**Table 1:** Supra-lexical word length, word frequencies, psycholinguistic rating values as well as semantic cohesion and number of different titles obtained for EAT-derived (EAT-1 to EAT-6) and matched (Match-1 to Match-6) items.

**Item**	**Length**	**Frequency^a^**	**Valence^b^**	**Arousal^b^**	**Imageability^b^**	**Cohesion^c^**	**Ntitle^d^**
Match-4	6	7,444	5.3	5.2	4.5	0.208	29
Match-1	5	33,772	5.7	5	5.1	0.225	27
Match-6	6.3	37,268	5.5	5.1	4.7	0.233	31
Match-5	4.6	27,688	5.1	5.1	4.9	0.239	26
Match-3	5	29,016	4.9	5.3	5	0.249	29
EAT-3	4.9	28,206	4.9	5.2	5.1	0.264	18
EAT-4	6.2	8,166	5.4	5.1	4.4	0.266	25
Match-2	5.7	80,781	5.8	5.2	4.8	0.266	31
EAT-1	5	33,902	5.7	5	5.1	0.289	20
EAT-2	5.6	82,912	5.7	5	4.8	0.296	26
EAT-6	6.3	38,108	5.5	5	4.6	0.337	20
EAT-5	4.7	28,270	5	5	5.1	0.369	16

a *Word frequencies were taken from van Heuven et al. (2014)*.

b *Psycholinguistic rating values were taken from Bestgen and Vincze (2012)*.

c *Intra-item semantic cohesion was determined as the grand mean of semantic similarity between each word of an item with all remaining other words of this item*.

d *Number of different titles found for each item*.

### 3.3. Measures for Semantic Similarities

We refer to the dependent variable as “semantic relatedness” in order to distinguish it from the independent variable “semantic cohesion.” In general, semantic similarity between two words is calculated based on a 300 dimensional vector space model trained with the fastText-based skipgram algorithm (Bojanowski et al., 2017) and based on the Gutenberg English Poetry Corpus (GEPC; Jacobs, 2018) using the similarity function of the gensim library (Řehůřek and Sojka, 2010) in Python 3.7.

#### 3.3.1. Determination of Semantic Relatedness

Semantic relatedness refers to our measure for the similarity between a title entered by the participants and all words of the corresponding micropoem. For each micropoem mean semantic similarity was calculated as individual mean of all semantic similarity values thus obtained. “Maths,” an element of the micropoem “proof” was not part of the GEPC corpus (nor were mathematics and all corresponding spellings). Therefore mean semantic relatedness for micropoem EAT-4 was calculated based on the remaining eight other words of the micropoem EAT-4.

#### 3.3.2. Determination of Semantic Cohesion

Semantic cohesion refers to the predictor variable and is assigned to the micropoems based on its construction only. It is determined as grand mean of semantic similarity between each word of a micropoem with all remaining other words of this micropoem (for individual semantic cohesion of the micropoems please see Table 1).

### 3.4. Data Collection

We introduced 12 items as examples of modern poetry to the participants. Because of the shortness, micropoems were presented as a vertically arranged list of words with one stanza (cf. Figure 1) and were displayed in random order. Participants were asked to give each micropoem a one-word title. Participants were explicitly instructed to avoid giving an element of the micropoem itself as a title. All titles given are summarised in Supplementary Tables 3, 4.

#### 3.4.1. Predictors

Besides the manipulation of semantic cohesion (*M* = 0.27, *SE* = 0.01, range = 0.21–0.31), we used the supra-lexical word frequency, the mean word frequency of all words constituting a micropoem as a second continuous predictor (*M* = 36294.42, *SE* = 6769.46, range = 7444–82912). The individual values for both predictors for all micropoems are reported in Table 1.

#### 3.4.2. Implicit Measures

Reaction times for first time reading as well as mean semantic relatedness between each title given and the corresponding words of each micropoem were collected as implicit measures for understandability of the micropoems presented. Reaction times were collected for first time reading of each micropoem. The time between loading of the page and the moment when the participant moved on to the next page was recorded. The precision of the measurement was 1 s. The reading time recorded for participant 143 and micropoem EAT-3 was 851 s. This is indicative of a temporary deflection of the participant's attention rather than a correct representation of the time taken to read the micropoem. Therefore the reading time for this particular micropoem and participant was approximated by the mean reading time of participant 143 for all other micropoems with high semantic relatedness.

#### 3.4.3. Explicit Measures

Additionally, participants were asked to rate their own reading experience on 5-point rating scales for dimensions Difficulty, Imagery (Kuiken et al., 2012), as well as Liking of the micropoem and experience of Mood (Lüdtke et al., 2014, cf. Table 2). This was used as an explicit measure for participants' reception of the poems. The order of the questions was randomised for each participant. Additionally, reading habits and individual preference for poetry (modern vs. classic) as well as enjoyment of poetry were assessed for each participant.

**Table 2:** Dimensions assessed and corresponding items in the poem questionnaire.

**Dimension**	**Item**	**References**
Liking	I like the poem.	Lüdtke et al., 2014
Experience of Mood	Reading the poem let me experience a mood.	Lüdtke et al., 2014
Imagery	While reading this poem the images that came to mind seemed pregnant with meaning.	Kuiken et al., 2012
Difficulty	I had to think long and hard about the poem before I could come up with a title.	This work

*Items were assessed on a 5-point rating scale*.

#### 3.4.4. Treatment of Invalid Titles

Titles for which the mean semantic relatedness value was not determined (either because the title entered was not part of the vocabulary or because it was a two-word title) were excluded in the linear mixed model analysis of semantic relatedness. This represents a 10.1% of the final dataset of all titles entered. All remaining titles were corrected for British orthography.

### 3.5. Statistical Analysis

A Box-Cox analysis using the MASS::boxcox() command (Venables and Ripley, 2002) incrementing by 0.1 within the interval [−6, 6] revealed a deviation from normal distribution of all dependent variables. The data was therefore transformed using the corresponding lambda (cf. Supplementary Table 5) and were used for inferential statistical analysis. Statistical analyses were conducted using linear mixed effects regression models (Baayen et al., 2008; Barr, 2013; Barr et al., 2013; Magezi, 2015) using the package lme4 (Bates et al., 2015) in the statistical environment R (R Core Team, 2020). Fixed effects of semantic cohesion and supra-lexical word frequency on dependent variables semantic relatedness of the titles, reading time, perceived liking, perceived mood, imagery experienced, perceived difficulty, and number of different titles found, respectively, were checked with Wald F-tests and a Kenward-Roger approximation of degrees of freedom using the car::Anova() function (Fox and Weisberg, 2019). Random effects were assumed for subjects and micropoem sets and were approximated allowing for individual intercepts for participants, and inclusion of random slopes where appropriate. In line with recent recommendations (Barr, 2013) complex models were generated for all variables (random slopes models and/or interaction models, where appropriate) and complex models were compared to models with sequentially decreased complexity to find the model fitting the data best. Model comparison was performed using the stats::anova() function. Unless otherwise indicated, the most parsimonious model, a linear mixed model with individual intercepts for random person and micropoem factors, was used for the final statistical analysis. A general linear model was used to predict the of number of different titles by semantic cohesion. This was calculated using the stats::lm() function. For all models, Nakagawa's *R*^*2*^ was calculated using the performance::r2_nakagawa function (Nakagawa and Schielzeth, 2013). All data were analysed on a Bonferroni-corrected alpha-level of 0.05/7 = 0.007. Please refer to Table 3 for a summary of the correlation matrix for the different dependent variables and to Table 4 for a summary of all final models.

**Table 3:** Correlation matrix of the dependent variables.

**Variable**	**1**	**2**	**3**	**4**	**5**	**6**	**7**
1. Mean semantic relatedness	-						
2. Reading times	−0.594	-					
3. Number of titles	−0.741	0.602	-				
4. Experience of Mood	0.545	−0.732	-0.507	-			
5. Liking	0.758	−0.788	−0.606	0.752	-		
6. Imagery	0.576	−0.866	−0.67	0.825	0.822	-	
7. Difficulty	−0.769	0.858	0.772	−0.629	−0.808	−0.869	-

**Table 4:** Summary of the statistical analysis.

**Variable**	**df_1_, df_2_**	***F***	***p***	**REM^a^**	***R*^2^ (conditional, marginal)**
Mean semantic relatedness	1, 9.92	27.6	0.0004	Intercepts	0.413, 0.248
Mean reading time	1, 10.11	8.09	0.0172	Intercepts	–
Number of titles^b^^,^ ^c^	1, 10	12.2	0.0058	n.a.	0.55, 0.504
Experience of mood	1, 10	4.45	0.06	Intercepts	–
Liking	1, 10	13.54	0.004	Intercepts	0.462, 0.039
Imagery	1, 10	5.04	0.048	Intercepts	–
Difficulty	1, 10	13.3	0.0045	Intercepts	0.401, 0.042

*Data were analysed on a Bonferroni-corrected alpha level of 0.05/7 = 0.007. Explicit and implicit measures are separated by a single line*.

a *Random effects modelling: intercepts = individual intercepts*.

b *A general linear model was employed and semantic cohesion was used as a predictor for the number of different titles found*.

c *R^2^ is given as multiple, adjusted*.

## 4. Results

We have always started our analysis with a complex model with maximal degrees of freedom for the random effects. All initial models contained both fixed effects, i.e., semantic cohesion and supra-lexical word frequency and their interaction (cf. Table 1). We then reduced the random effects model in stepwise manner until the model was no longer overfitting (Baayen et al., 2008; Barr, 2013; Barr et al., 2013). For all dependent variables interaction or main effects with supra-lexical word frequency never became significant (all *F* < 0.4, all *p* > 0.5) and were therefore removed.

### 4.1. Objective Measures

**Figure 2:**
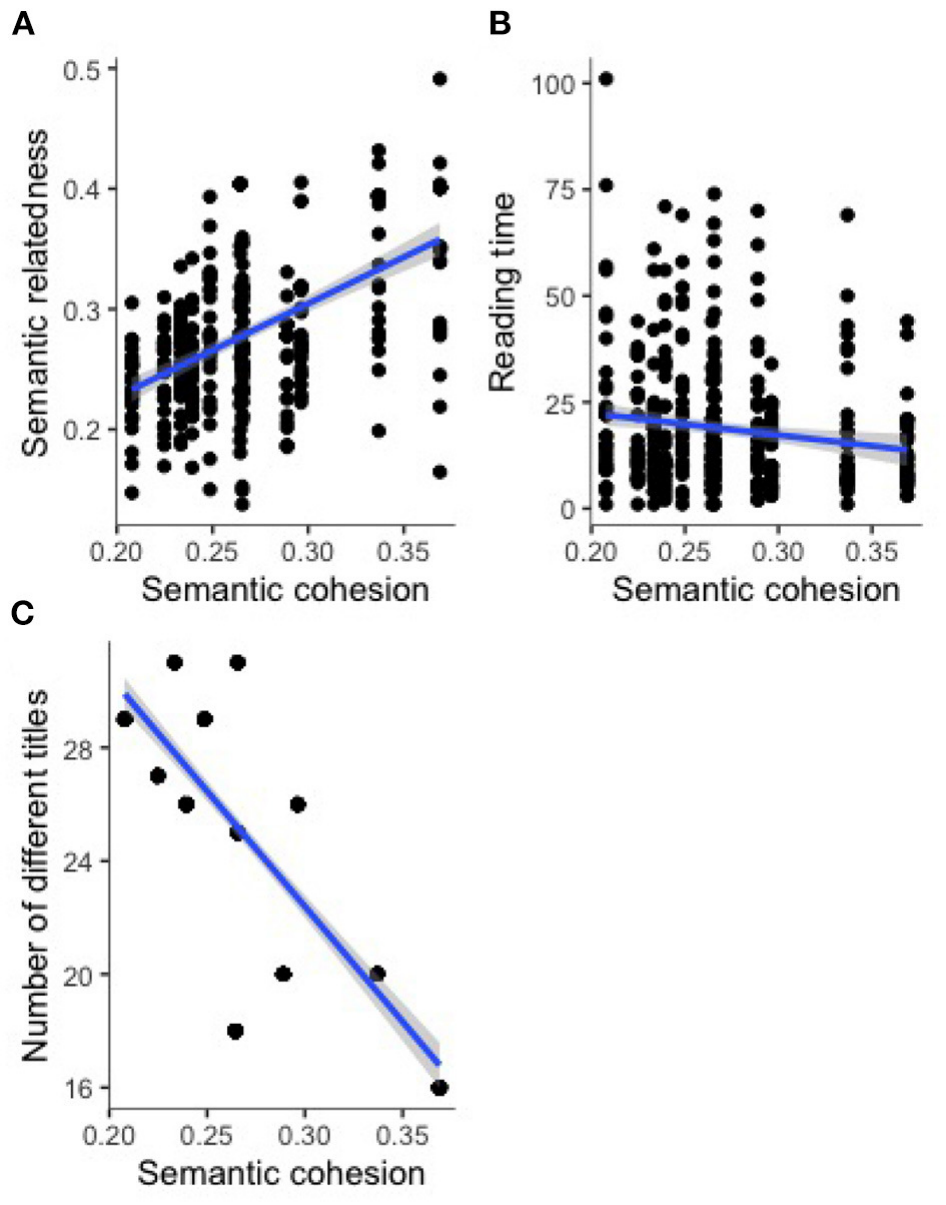
Explicit measures: Relationship between semantic cohesion and semantic relatedness between titles and poems **(A)**, mean reading time **(B)**, and number of different titles **(C)**.

A positive relationship between semantic cohesion and semantic relatedness as a proxy for the closing of a good Gestalt (cf. Figure 2A) revealed to be significant (cf. Table 4, entry 1). A slightly negative relationship was observed between mean reading time and semantic cohesion (cf. Figure 2B) but did not become significant on a Bonferroni-corrected alpha level (cf. Table 4, entry 2). A strongly negative relationship between semantic cohesion and number of different titles (cf. Figure 2C) revealed to be significant on the Bonferroni-corrected alpha-level (cf. Table 4, entry 3).

### 4.2. Subjective Measures

**Figure 3:**
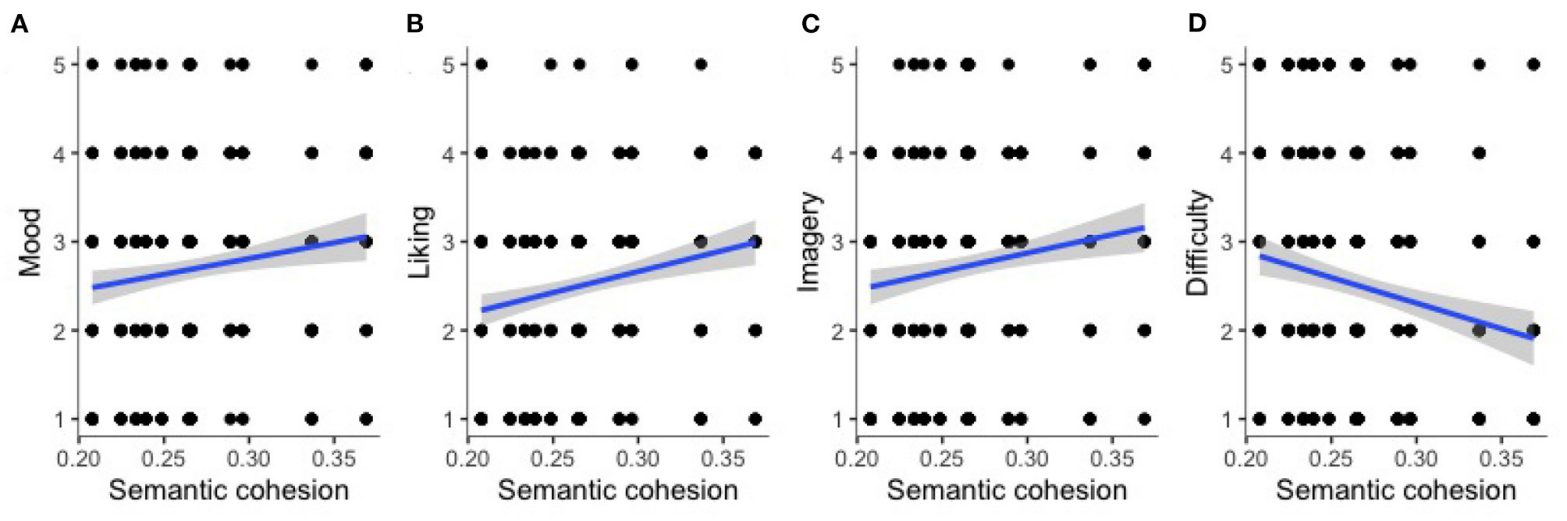
Implicit measures: Relationship between semantic cohesion and participants rating of perceived mood **(A)**, liking of the items **(B)**, imagery **(C)**, and perceived difficulty in understanding **(D)**.

A moderately positive relationship between semantic cohesion and participants' reported perception of mood (cf. Figure 3A) did not reveal to be significant on the Bonferroni-corrected alpha-level (cf. Table 4, entry 4). A moderately positive relationship was found between semantic cohesion and participants' reported liking of the items (cf. Figure 3B) and was significant on the Bonferroni-corrected alpha-level (cf. Table 4, entry 5). Moreover, the moderately positive relationship between semantic cohesion and participants' reported perception of mental images (cf. Figure 3C) did not reveal to be significant on the Bonferroni-corrected alpha-level (cf. Table 4, entry 6), while the negative relationship between semantic cohesion and participants' reported perception difficulty of understanding the items (cf. Figure 3D) was significant on the Bonferroni-corrected alpha-level (cf. Table 4, entry 7).

## 5. Discussion

We have set out to understand the mechanisms that make us, as readers of a poem, see a bigger picture upon the inward eye. We hypothesised that even background features should have a decisive influence on reading and understanding poetic texts. More precisely, we assumed that semantic relatedness of the words making up a poem, as one example for a supra-lexical background feature, is a main factor governing the formation of the closed Gestalt. To test this hypothesis we had participants read lists of words with different amount of semantic relatedness and assayed their emotional response as well as their associations as a proxy for the mental images produced. Moreover we also manipulated supra-lexical word frequency defined as the mean word frequency of all words composing the micro poem, to test whether possible effects rely on a simple facilitation of word identification only. Taken together, we found no effect for supra-lexical word frequency but an effect for semantic cohesion on all objective variables as well as on subjective ratings liking and difficulty.

The observed differences of semantic relatedness between titles and micro-poems as a function of semantic cohesion lets us understand two things: first we can safely conclude that our manipulation was successful. Secondly, on condition that semantic relatedness of the titles is indicative of the capacity of a poem to induce a mental image, we have shown that the closing of a good Gestalt is at least partially hinged on semantic cohesion of a micropoem. Up to this point a mere effect of facilitated processing would be an equally plausible explanation for the present results. If that was the case, however, supra-lexical word frequency, which famously facilitates processing of words and texts (Lüdtke et al.)^2^ should lead to the same results. We, however, did not find any main or interaction effect of word frequency on any variable examined. Therefore the present results need to be explained through effects beyond facilitation of processing. Our findings are in line with the results of Jolsvai et al. (2013), showing that meaningfulness of sentence fragments is a superior predictor for language processing speed than word frequency. Taken together this indicates that regarding the formation of a bigger picture facilitation of lexical access, i.e., basic comprehension processes at simple word level, is a second player to integration processes. In the following we will discuss this in more detail in reflection of current discourse on mental imagery, aesthetic appreciation, and comprehension difficulty.

Let us begin with an analysis of the results concerning mental imagery. Further to semantic relatedness, we analysed the number of different titles between items with high and low semantic cohesion, as a direct measure of mental imagery and the closing of a good Gestalt. Items with high semantic cohesion were expected to point to a target word or induce a target concept and therefore to yield a lower number of different associations. Items with low semantic cohesion on the other hand should lead to the generation of more different and less related associations. The present data confirmed this hypothesis: items with high semantic cohesion yielded fewer and less semantically related associations. While the analysis of the number of titles would indicate that a good Gestalt was indeed derived from items with high semantic cohesion, directly assayed generation of mental imagery (see above) did not. It can only serve as indicator or a trend pointing in the same direction. Although the latter finding at first glance seems to contradict our hypothesis, it is, upon closer inspection, a faithful reflection of the ongoing discussion whether mental imagery is indeed produced during reading, or is more of a literal afterthought (Jacobs and Willems, 2018). Moreover, the poems were essentially devoid of a deeper meaning or plot (Teng et al., 2016). This already by and in itself may disturb the process of closing a good Gestalt and thereby have interfered with the creation of mental images. In fulfillment of Rosenblatt's Transactional Theory (Rosenblatt, 1978), we also may not neglect the reader in this equation. Reader-based variables, particularly their response to the set task and the consequently adopted reading goal, can increase a reader's tendency to employ an inferential reading mode (Bohn-Gettler, 2019). In light of this, the task set here may have distracted the reader from adopting an inferential reading mode which otherwise would have allowed for mental images to be drawn. The readers may instead have focussed on *getting it right* and may therefore have lost access to any actively communicable notion of a mental image. Looking at participants' ratings for aesthetic appreciation of the items, no effects were found on dimension experience of Mood, but clear effects were obtained for Liking. This is in opposition to the predictions derived from the NCPM, according to which manipulation of background features should be reflected in upper route processes more than in lower route processes. Back- and foregrounding elements, however, cannot be thought as entities isolated from each other, seen as they mutually affect each other (Reber et al., 2004). In this sense, Obermeier et al. (2013) showed that manipulation of foreground features can influence upper route processes. Also Lüdtke et al. (2014) demonstrated cross-communication between foregrounding and backgrounding elements. These findings point in the direction already outlined by Jacobs (2011) suggesting that it is the quotient of backgrounding and foregrounding elements more than their individual contribution that facilitates enhanced processing along either route. More research is needed to shed light on this effect. In this sense, our approach lends itself to the analysis of the influence of semantic activation on both trajectories through the use of a set of micropoems with continuously increasing semantic cohesion. Moreover Belfi et al. (2018) showed that vividness of mental imagery has a crucial effect on aesthetic appreciation of poems.

On the axis Comprehensability, the subjective measure (survey data) became significant, while the objective measure (reading time) just failed to be significant on a Bonferroni corrected alpha level, but still indicated a correlation on a descriptive level. Reading time could, theoretically, be construed as a measure for ease of processing. By showing that word frequency, a factor directly related to processing speed, does not have any influence on reading times we demonstrate that semantic cohesion does not only influence basic language processing like word identification but presumably also higher order processes like integration and the closing of a good Gestalt. We argue that micropoems encouraging the formation of mental images should be easier understood and hence read faster. Magyari et al. (2020), per contra, reported prolonged fixation duration on words associated with an enactive style text, for which participants reported more mental images. On a fist glance this, again, seems to contradict our results which indicated faster reading times for items that were reported to induce more mental images. Yet, processing a prose text differs fundamentally from processing of our micropoems. Reading poetic texts is characterised by enhanced re-reading (Xue et al., 2020). Therefore the reading times measured in this study not only reflect ongoing reading processes (i.e., first pass reading and fixation duration as captured in the eyetracking data reported by Magyari et al., 2020) but also higher order comprehension processes necessary for fulfilling the task at hand. Contrary to studies on literary reading, in our case subjects had to find one word titles. We assumed that semantic cohesion fosters the closing of a good Gestalt which leads to enhanced mental imagery. This should simplify the task and thereby enhance participants' reaction times. More studies need to sheet light onto the role of mental imagery in reading prose and poetry. The method employed here is a promising tool to account for factors influencing mental imagery when reading poetic texts.

### 5.1. Limitations and Outlook

The Edinburgh Associative Thesaurus, which we used as database for the generation of our items with high semantic cohesion was created in the 1970ies and therefore may not fully reflect the current-day associative landscape. The use of recent computational tools such as fastText (Bojanowski et al., 2017) or GloVe (Pennington et al., 2014) in combination with a modern corpus is a preferable choice for the generation of items with high semantic cohesion. The current data show that semantic cohesion influences the generation of associations as well as liking (and possibly by extension also mood and mental imagery) through mechanisms which exceed mere facilitation of processing. Yet, our data show that high semantic cohesion does not automatically lead to the generation of mental images. As much as we expect emotions to be dependent on text, task, and reader (Rosenblatt, 1978; Bohn-Gettler, 2019) we may claim the same to be true for mental imagery. This should be considered in the item construction and design of future studies in order to disentangle the individual influences of person-, text-, and task-related factors on the formation of mental images. Moreover the perceived quality or meaningfulness of the items employed should be verified in a pre-study.

On a formal-methodological account, the method established in this work is well-suited to study the much-discussed question of when and where mental images are generated during the reading of poems. Our items were specifically designed to serve as a “minimal example” of a poem, which is devoid of all features of a traditional poem except one: semantic cohesion. This was deliberately done to minimise confounding effects through other variables traditionally present in poetry. The use of such items facilitated the isolated analysis of the effect of semantic relatedness on the closing of a good Gestalt. Moreover the items, devoid by design of foregrounding elements, gave us a tool to singly manipulate background features. Our results demonstrated that simple manipulation of background features is possible in a set-up with short stimuli. Our method is therefore relevant for fMRI studies, which can help to explore the assumed higher order processes during online-formation of mental imagery.

What is more, our method is particularly tailored to manipulate and target the complex interaction between background features and text processing along the upper and lower route postulated by the NCPM. This ultimately allows to conceptualise backgrounding as more than an instance from which literary devices called foregrounding elements stand out (Hakemulder, 2020).

Nevertheless, the effects observed in this study ought to be verified using longer items with more stylistic elements normally associated with poetic texts such as rhyme schemes, meter and verse form. Based on our findings we expect also higher appreciation and better understanding for more poem-like material with high semantic cohesion compared to low cohesion. We instructed our participants to treat our items as an example of modern poetry, which should have an influence on the reading mode, as for example, demonstrated by the fact-fiction paradigm (Altmann et al., 2014). Yet based on the current data we cannot rule out that some participants interpreted our items as arbitrary word lists instead of poems. Future studies using fMRI might answer that question: If the micropoems were processed as poetic texts, activation in the bilateral precentral and inferior frontal gyrus, as well as the right dorsolateral prefrontal cortex, anterior insula, and temporal pole should be observed (Jacobs and Willems, 2018).

### 5.2. Conclusion

Each and any of our analyses leads to the conclusion that associations, although they may have an influence on early processes, shape and guide higher integration processes. We therefore suggest that the bigger picture, as it were, of a poem is coloured by a collection of associations.

Psycholinguistic elements of a text amalgamate to create something larger which we, as readers, experience as *a poem*. We have shown that the synthesis of a poem from its elements exceeds the effect of facilitated processing. Significantly higher reported liking of items with high semantic cohesion and reduced perceived difficulty in understanding of the same items in combination with the respective reduced number of different titles strongly suggests that semantic cohesion influences the closing of a good Gestalt and as such may catalyse the generation of mental imagery. Although items with high semantic cohesion, devoid of meaning and plot, may not be sufficient to fully induce a mood and create communicable mental images, we have shown that they do pave the path toward the generation of mental images.

## Data Availability Statement

The raw data supporting the conclusions of this article will be made available by the authors, without undue reservation.

## Ethics Statement

The studies involving human participants were reviewed and approved by Ethik Kommission der Freien Universität Berlin. The patients/participants provided their written informed consent to participate in this study.

## Author Contributions

KH and JL contributed equally to the conception and design and report of the study. All authors have read and agreed to the final version of the manuscript.

## Funding

We acknowledge support by the Open Access Publication Fund of the Freie Universität Berlin.

## Conflict of Interest

The authors declare that the research was conducted in the absence of any commercial or financial relationships that could be construed as a potential conflict of interest.

## Publisher's Note

All claims expressed in this article are solely those of the authors and do not necessarily represent those of their affiliated organizations, or those of the publisher, the editors and the reviewers. Any product that may be evaluated in this article, or claim that may be made by its manufacturer, is not guaranteed or endorsed by the publisher.

## Footnotes

^1^We realised that in one case the EAT-based micropoem EAT-3 and its corresponding Match-based micropoem Match-3 both contained one identical word (“love”).

^2^Lüdtke, J., Fröhlich, E., and Jacobs, A. M. (submitted). Small words matter: An eyetracking study about reading affective words in context. *Front Psychol*.
