# Supplementary material for: Micropoetry Meets Neurocognitive Poetics: Influence of Associations on the Reception of Poetry
Source: Front Psychol. 2021 Oct 20;12:737756. doi: 10.3389/fpsyg.2021.737756 (PMC8563571; doi:10.3389/fpsyg.2021.737756)
Supplement: Supplementary file 1 [file Data_Sheet_1.PDF]

frontiers

Supplementary Material

1 SUPPLEMENTARY DATA

Table S1:
Word frequencies (van Heuven et al., 2014) and psycholinguistic rating values (Bestgen and Vincze, 2012) for all EAT-derived items presented in this study.

Words	Length	Frequency	Valence	Arousal	Imagery	Dominance	Concreteness
EAT-1							
house	5	135288	5.48	4.41	5.42	5.02	5.44
guy	3	28510	4.69	5.49	4.55	4.89	4.04
girl	4	39601	6.53	5.4	5.26	5.45	4.72
baby	4	39312	6.06	5.02	5.3	5.18	5.03
bird	4	14348	5.7	4.83	5.61	5.21	6.05
rag	3	777	5.59	4.82	5.61	5.42	5.79
child	5	28117	5.5	5.16	4.64	5.06	3.78
childhood	9	4080	5.45	5.37	4.1	4.99	3.07
china	5	15085	6.01	4.37	5.62	5.28	5.89
EAT-2							
past	4	39888	5.82	5.24	4.27	5.41	3.58
present	7	16045	6.13	5.07	3.33	5.59	2.96
time	4	451369	5.85	5.24	4.22	5.4	3.57
life	4	131194	5.42	5.22	4.33	5.16	3.09
bright	6	13304	6.54	4.77	5.76	5.34	5.05
ahead	5	28309	5.56	5.08	5.04	5.21	4.83
black	5	36769	4.67	4.78	5.24	4.88	4.96
blue	4	29263	6.16	4.48	5.75	5.28	5.69
clairvoyant	11	64	5.52	5.17	5.3	5.16	4.58
EAT-3							
transplant	10	1329	4.06	5.26	4.99	4.47	4.92
beat	4	23766	5.31	5.82	5.1	5.19	4.49
love	4	151607	5.71	5.68	4.78	5.3	3.3
soul	4	6776	4.78	5.28	4.67	4.97	3.34
ache	4	453	4.87	4.68	5.22	4.7	4.77
throb	5	116	4.55	5.17	5.33	4.61	4.66
attack	6	15860	3.72	6.18	4.98	4.76	4.71
pump	4	2590	5.27	4.6	5.09	5	5.1
red	3	51355	5.84	4.48	5.63	5.23	5.79
EAT-4							
evidence	8	23259	4.67	5.16	3.53	4.8	3.20
pudding	7	5543	6	4.46	5.93	5.22	6.32
whisky	6	2498	4.53	5.25	5.13	4.75	4.85

Continued on next page

Table S1 – continued from previous page

Words	Length	Frequency	Valence	Arousal	Imagery	Dominance	Concreteness
theory	6	5599	6.21	4.96	3.63	5.59	3.16
copy	4	4824	5.59	4.51	4.88	5.21	5.07
detective	9	3627	4.09	5.7	4.54	4.48	3.96
identity	8	4510	6.06	5.72	3.86	5.62	3.02
law	3	22552	4.62	4.94	3.91	4.74	3.52
maths	5	1086	6.85	5.26	4.3	6.01	3.77
EAT-5							
bed	3	26282	5.51	4.4	5.47	4.87	5.22
dream	5	19951	6.19	5.22	5.17	5.36	3.89
awake	5	2409	5.53	4.45	5.59	4.87	4.8
night	5	90177	5.54	4.52	5.57	4.83	4.72
rest	4	38188	5.23	4.7	4.58	4.96	4.05
peace	5	11159	4.69	5.95	4.47	5.22	3.82
sound	5	29460	5.06	4.84	5.08	4.85	4.69
tired	5	8859	4.89	4.89	5.04	4.75	4.31
death	5	27948	2.8	5.77	4.79	3.93	3.54
EAT-6							
matter	6	35202	5.76	5.12	4.09	5.4	3.81
material	8	7472	5.89	4.62	4.54	5.4	4.78
chemical	8	2615	4.14	4.99	4.44	4.69	4.65
thing	5	175602	5.75	5.55	4.18	5.52	3.61
stuff	5	49420	4.84	5.32	4.8	4.97	4.38
chemistry	9	1801	5.97	5.02	4.28	5.42	4.16
element	7	5764	5.58	4.71	4.32	5.14	4.71
food	4	56471	5.98	4.73	5.44	5.31	5.58
metal	5	8629	5.61	4.58	5.24	5.26	5.9

Note: Items are stated in italics above each group of words making up the items.
Items are visually grouped by a dotted line.

Table S2:
Word frequencies (van Heuven et al., 2014) and psycholinguistic rating values (Bestgen and Vincze, 2012) for matched items presented in this study.

Words	Length	Frequency	Valence	Arousal	Imagery	Dominance	Concreteness
Match-1							
money	5	138993	5.73	5.36	4.69	5.42	4.42
wife	4	28183	5.7	5.39	4.92	5.16	4.15
light	5	38200	6.12	4.66	5.66	5.22	5.1
film	4	36996	6.04	5.21	4.94	5.34	5.07
cream	5	15576	5.74	4.49	6	5.14	6.31
ewe	3	513	5.15	4.64	5.76	4.97	6.05

Continued on next page

Table S2 – continued from previous page

Words	Length	Frequency	Valence	Arousal	Imagery	Dominance	Concreteness
tough	5	26446	4.9	5.43	4.63	5.15	4.24
fortunate	9	2323	6.01	5.31	4.12	5.56	3.07
field	5	16714	5.81	4.76	5.38	5.32	5.63
Match-2							
bank	4	38406	5.25	5.32	4.23	5.02	4.1
ability	7	8807	6.46	5.36	3.77	5.84	3.12
good	4	454018	6.31	5.46	4.08	5.6	3.45
sure	4	125797	5.66	5.35	4.41	5.24	3.74
castle	6	9715	6.62	5.3	5.29	5.91	5.2
match	5	26788	5.72	5.57	5.11	5.38	4.96
piece	5	37552	5.01	4.43	5.25	4.86	5.53
cold	4	25839	5.7	4.59	5.75	5.08	5.22
astonishment	12	108	5.24	5.32	4.94	5.14	4.49
Match-3							
relaxation	10	452	4.12	5.45	4.55	4.52	4.18
fast	4	24850	5.23	5.51	4.95	5.27	4.61
love	4	151607	5.71	5.68	4.78	5.3	3.3
evil	4	5670	4.77	5.62	4.46	4.87	3.19
lazy	4	2381	4.85	5.01	5.11	4.79	4.82
flame	5	2236	4.38	5.36	5.41	4.8	5.03
battle	6	17431	4.63	6.01	5.13	5.21	4.95
hose	4	716	5.28	4.45	4.94	5.05	5.1
face	4	55804	5.43	5.04	5.25	5.16	4.89
Match-4							
result	6	23390	4.73	5.33	3.38	4.79	3.16
banana	6	3304	5.94	4.46	5.86	5.24	6.29
fierce	6	1891	4.3	5.81	4.95	4.78	4.44
active	6	4316	5.78	5.57	3.65	5.37	3.43
tape	4	4419	5.44	4.59	4.99	5.13	5.3
cigarette	9	2568	3.75	5.02	4.8	4.38	4.72
equality	8	1606	5.63	5.2	3.88	5.55	2.97
poor	4	22377	5.07	5.02	4.42	5.07	3.51
charm	5	3128	6.8	5.49	4.71	5.77	3.51
Match-5							
hot	3	28098	5.75	4.56	5.9	5.15	5.79
score	5	21653	6.1	5.26	4.75	5.62	4.39
curve	5	1633	5.36	4.32	4.99	4.99	4.96
head	4	81979	5.05	4.92	5.32	4.95	5.3
bank	4	38406	5.25	5.32	4.23	5.02	4.1
alive	5	13388	4.55	5.27	4.66	4.56	4.01
tough	5	26446	4.9	5.43	4.63	5.15	4.24
noise	5	12039	4.88	5.45	5.31	4.84	4.7

Continued on next page

Table S2 – continued from previous page

Words	Length	Frequency	Valence	Arousal	Imagery	Dominance	Concreteness
court	5	25553	4.22	5.01	4.09	4.48	3.8
Match-6							
friend	6	38876	5.56	5.54	4.53	5.16	3.56
magazine	8	3903	6.2	4.98	4.65	5.39	4.45
cigarette	9	2568	3.75	5.02	4.8	4.38	4.72
quite	5	165758	5.59	5.17	4.04	5.17	3.26
news	4	51855	5.6	5.54	4.64	5.13	4.27
infertile	9	103	5.51	5.45	4.5	5.26	4.14
cabinet	7	8795	5.75	4.93	4.34	5.64	4.16
face	4	55804	5.43	5.04	5.25	5.16	4.89
grass	5	7748	5.69	4.29	5.54	5.03	5.76

Note: Items are stated in italics above each group of words making up the items.
Items are visually grouped by a dotted line.

Table S3:
Complete list of title words given by the participants and corresponding semantic relatedness values for EAT-derived items

Title	Semantic relatedness
EAT 1	
1950s	NA
Corvid	NA
growing-up	NA
suburbia	NA
tree house	NA
stereotypes	NA
family life	NA
dolls	0.331
room	0.311
home	0.287
home	0.287
home	0.287
home	0.287
home	0.287
family	0.278
family	0.278
family	0.278
family	0.278
family	0.278
family	0.278
family	0.278

Continued on next page

Table S3 – continued from previous page

Title	Semantic relatedness
family	0.278
family	0.278
family	0.278
family	0.278
life	0.265
charm	0.262
play	0.260
marriage	0.237
marriage	0.237
poverty	0.226
floors	0.225
affair	0.212
relationship	0.206
limbo	0.201
adoption	0.187
adoption	0.187
growing	0.185
EAT 2	
senitude	NA
timeline	NA
psycho	NA
mystique	NA
time	0.405
vision	0.390
vision	0.390
always	0.323
thoughts	0.319
future	0.318
future	0.318
future	0.318
future	0.318
future	0.318
future	0.318
future	0.318
future	0.318
generations	0.315
foresight	0.301
soul	0.297
meaning	0.279
feeling	0.277
reminiscence	0.275
memory	0.272

Continued on next page

Table S3 – continued from previous page

Title	Semantic relatedness
tides	0.272
mystery	0.271
roundabout	0.267
narrative	0.266
flying	0.266
stick	0.263
mystic	0.259
prophet	0.251
witch	0.247
involved	0.245
travel	0.237
flow	0.229
temporal	0.226
magic	0.222
EAT 3	
TERM	NA
heart	0.404
heart	0.404
heart	0.404
heart	0.404
heart	0.404
heart	0.404
heart	0.404
heart	0.404
heart	0.404
heart	0.404
heart	0.404
heart	0.404
heart	0.404
heart	0.404
heart	0.404
heart	0.404
pain	0.352
pain	0.352
hearts	0.346
transplant	0.318
anger	0.307
operation	0.301
connection	0.280
music	0.279
music	0.279
illness	0.267

Continued on next page

Table S3 – continued from previous page

Title	Semantic relatedness
agony	0.260
matador	0.234
heartfelt	0.233
relationship	0.231
sanguine	0.222
surgery	0.221
murder	0.220
medicine	0.214
claws	0.200
hospital	0.191
survival	0.181
EAT 4	
tv	NA
murder-mystery	NA
Goertzel	NA
CSI	NA
police station	NA
summerschool	NA
crime novel	NA
proof	0.360
proof	0.360
detective	0.356
traditional	0.325
trial	0.316
science	0.309
crime	0.299
crime	0.299
crime	0.299
cop	0.290
mystery	0.283
murder	0.271
murder	0.271
mystery	0.270
forensics	0.266
cutthroat	0.253
university	0.252
confusion	0.240
film	0.235
film	0.235
Sherlock	0.234
Sherlock	0.234
Sherlock	0.234

Continued on next page

Table S3 – continued from previous page

Title	Semantic relatedness
Sherlock	0.234
play	0.231
lessons	0.223
wasted	0.207
noir	0.197
Bosch	0.153
flock	0.149
Barnaby	0.138
EAT 5	
night terrors	NA
rest	0.491
sleeping	0.421
slumber	0.404
sleep	0.401
sleep	0.401
sleep	0.401
sleep	0.401
sleep	0.401
sleep	0.401
sleep	0.401
sleep	0.401
sleep	0.401
sleep	0.401
sleep	0.401
sleep	0.401
sleep	0.401
sleep	0.401
sleep	0.401
sleep	0.401
life	0.352
life	0.352
life	0.352
twilight	0.349
twilight	0.349
tranquil	0.342
serenity	0.340
aging	0.339
finally	0.331
calm	0.289
insomnia	0.284
beginning	0.282
dark	0.278

Continued on next page

Table S3 – continued from previous page

Title	Semantic relatedness
farewell	0.260
ghost	0.245
cycle	0.219
relax	0.219
staccato	0.165
EAT 6	
Gerunch	NA
additives	NA
bowtie	NA
CERN	NA
substance	0.432
elements	0.421
science	0.394
science	0.394
science	0.394
science	0.394
science	0.394
science	0.394
science	0.394
science	0.394
science	0.394
science	0.394
expanse	0.394
physics	0.394
physics	0.394
science	0.394
science	0.394
science	0.394
processes	0.387
molecular	0.363
work	0.360
reaction	0.337
living	0.320
nouns	0.319
new	0.317
atom	0.301
innovation	0.291
science	0.281
scientist	0.276
scientist	0.276
atoms	0.266
lab	0.249

Continued on next page

Table S3 – continued from previous page

Title	Semantic relatedness
science	0.227
cooking	0.199

Note: Semantic relatedness between titles and poems was calculated as the mean similarity between titles given and individual words of each poem.
Similarities were calculated based on a 300 dimensional vector space model trained with the fastText-based skipgram algorithm (Bojanowski et al., 2017) and based on the Gutenberg English Poetry Corpus (Jacobs, 2018) using the similarity function of the gensim library (Řehůřek and Sojka, 2010) in Python 3.7.
Words that were not part of the GEPC were excluded from this calculation (NA).

Table S4:
Complete list of title words given by the participants and corresponding semantic relatedness values for Match-derived items

Title	Semantic relatedness
Match 1	
random shit	NA
the 60s	NA
moisturizer	NA
breadwinner	0.348
provider	0.310
sheep	0.291
milking	0.285
farm	0.275
seventies	0.274
farmer	0.271
farmer	0.271
farmer	0.271
countryside	0.271
life	0.267
life	0.267
life	0.267
farming	0.266
home	0.265
home	0.265
daily	0.256
spring	0.256

Continued on next page

Table S4 – continued from previous page

Title	Semantic relatedness
power	0.253
over	0.249
femininity	0.247
wedding	0.233
possibility	0.232
pastime	0.231
confusion	0.226
female	0.225
divorce	0.215
pastoral	0.203
feminine	0.201
golf	0.195
allegory	0.194
Wales	0.187
cinema	0.184
random	0.169
random	0.169
Match 2	
?	NA
break-in	NA
HSBC	NA
surprise	0.354
success	0.337
strength	0.320
work	0.318
king	0.317
security	0.312
security	0.312
judgement	0.309
checkmate	0.306
marble	0.289
nobility	0.286
worth	0.283
weakness	0.280
safety	0.271
capable	0.269
nothing	0.266
gold	0.264
market	0.263
rich	0.257
wealth	0.256
achievement	0.256

Continued on next page

Table S4 – continued from previous page

Title	Semantic relatedness
holding	0.251
career	0.251
accomplish	0.249
pressure	0.249
unknown	0.245
lottery	0.244
strife	0.237
random	0.232
random	0.232
class	0.231
buildings	0.230
robbery	0.222
concrete	0.212
rocket	0.210
Match 3	
firehouse	NA
PlayStation	NA
firefighter	NA
firefighter	NA
hose	0.393
love	0.369
fight	0.347
time	0.331
fire	0.329
fire	0.329
fire	0.329
war	0.325
war	0.325
intense	0.317
forest	0.315
conflict	0.309
mood	0.303
opposition	0.293
friendship	0.289
feeling	0.288
emotion	0.281
summer	0.278
dream	0.278
devil	0.274
passing	0.265
gardening	0.263
city	0.261

Continued on next page

Table S4 – continued from previous page

Title	Semantic relatedness
sleep	0.256
vacation	0.253
changes	0.251
bonfire	0.236
relationship	0.224
relationships	0.219
irritate	0.216
contemporary	0.215
arson	0.204
Henry	0.176
crazy	0.150
Match 4	
topsy-turvy	NA
activism	NA
fairtrade	NA
heirarchy	NA
bus stop	NA
resistance	0.305
gender	0.280
sport	0.278
action	0.276
more	0.275
competition	0.274
competition	0.274
disadvantaged	0.268
unconnected	0.260
work	0.258
confusion	0.254
confusion	0.254
class	0.253
fruit	0.252
fruit	0.252
what	0.244
efforts	0.242
discrepancy	0.240
student	0.233
life	0.232
homeless	0.231
police	0.228
news	0.228
random	0.225
random	0.225

Continued on next page

Table S4 – continued from previous page

Title	Semantic relatedness
book	0.220
monkey	0.211
islands	0.210
camp	0.208
Castro	0.201
stoned	0.181
running	0.171
Bahamas	0.147
Match 5	
shit	NA
Nascar	NA
head	0.342
track	0.308
tough	0.307
business	0.300
pain	0.284
stressful	0.282
car	0.277
young	0.272
contour	0.268
race	0.260
life	0.257
judgement	0.251
wooden	0.247
volleys	0.242
competition	0.241
sports	0.233
frustration	0.230
music	0.230
balls	0.228
sigh	0.228
tennis	0.224
tennis	0.224
tennis	0.224
tennis	0.224
tennis	0.224
tennis	0.224
tennis	0.224
tennis	0.224
tennis	0.224
food	0.219
games	0.213

Continued on next page

Table S4 – continued from previous page

Title	Semantic relatedness
pursuit	0.212
lawyer	0.211
crime	0.210
Wimbledon	0.196
urban	0.168
Match 6	
socialise	NA
newsagent	NA
Instagram	NA
socialise	NA
friends	0.335
woman	0.303
chat	0.298
conversation	0.295
communication	0.291
night	0.286
life	0.281
chatting	0.278
identity	0.278
family	0.276
home	0.273
strangeness	0.269
shock	0.263
smoking	0.255
health	0.253
springtime	0.249
conversation	0.249
sorrow	0.244
lost	0.243
tragedy	0.240
loss	0.240
cold	0.239
shop	0.236
uncertainty	0.235
despair	0.235
gossip	0.231
summer	0.230
bar	0.229
single	0.227
random	0.212
random	0.212
switch	0.203

Continued on next page

Table S4 – continued from previous page

Title	Semantic relatedness
growth	0.191
model	0.188

Note: Semantic relatedness between titles and poems was calculated as the mean similarity between titles given and individual words of each poem.
Similarities were calculated based on a 300 dimensional vector space model trained with the fastText-based skipgram algorithm (Bojanowski et al., 2017) and based on the Gutenberg English Poetry Corpus (Jacobs, 2018) using the similarity function of the gensim library (Řehůřek and Sojka, 2010) in Python 3.7.
Words that were not part of the GEPC were excluded from this calculation (NA).

General R script for Box-Cox transformation of a variable (adapted from Mangiafico, 2016):

```
> Box_var = boxcox(data$variable ~ 1,
                      lambda = seq(-6,6,0.1)
 )
> Cox_var = data.frame(Box_var$x, Box_var$y)
> Cox2_var = Cox_var[with(Cox_var, order(-Cox_var$Box_var.y)),]
> Cox2_var[1,]
> lambda = Cox2_var[1, "Box_var.x"]
> T_box_var = (data$variable ^ lambda - 1)/lambda # transformed variable
> # note: if lambda == 0: T_box_var = log(var)
```

Table S5:
Extracted lambdas for Box-Cox transformation

Variable	Lambda
mean semantic relatedness	0
reading time	0.2
Liking of the item	0.8
Induction of Mood	0.7
Imagery	0.8
Difficulty	0.1

REFERENCES

Bestgen, Y. and Vincze, N. (2012). Checking and bootstrapping lexical norms by means of word similarity indexes. *Behavior Research Methods* 44, 998–1006

Bojanowski, P., Grave, E., Joulin, A., and Mikolov, T. (2017). Enriching Word Vectors with Subword Information. *Transactions of the Association for Computational Linguistics* 5, 135–146

Jacobs, A. M. (2018). The Gutenberg English Poetry Corpus: Exemplary Quantitative Narrative Analyses. *Frontiers in Digital Humanities* 5, 1–14

Mangiafico, S. S. (2016). Summary and Analysis of Extension Program Evaluation in R, version 1.18.8.

Řehůřek, R. and Sojka, P. (2010). Software Framework for Topic Modelling with Large Corpora. In *Proceedings of the LREC 2010 Workshop on New Challenges for NLP Frameworks* (Valletta, Malta: ELRA), 45–50

van Heuven, W. J., Mandera, P., Keuleers, E., and Brysbaert, M. (2014). SUBTLEX-UK: A new and improved word frequency database for British English. *Quarterly Journal of Experimental Psychology* 67, 1176–1190
